# Association of Traditional Chinese Medicine Body Constitution and Health-Related Quality of Life in Female Patients with Systemic Lupus Erythematosus: A Cross-Sectional Study

**DOI:** 10.1155/2021/5568219

**Published:** 2021-07-22

**Authors:** Ning-Sheng Lai, Ming-Chi Lu, Hsiu-Hua Chang, Hui-Chin Lo, Chia-Wen Hsu, Kuang-Yung Huang, Chien-Hsueh Tung, Bao-Bao Hsu, Cheng-Han Wu, Malcolm Koo

**Affiliations:** ^1^Division of Allergy, Immunology and Rheumatology, Dalin Tzu Chi Hospital, Buddhist Tzu Chi Medical Foundation, Dalin, Chiayi, Taiwan; ^2^School of Medicine, Tzu Chi University, Hualien City, Taiwan; ^3^Department of Medical Research, Dalin Tzu Chi Hospital, Buddhist Tzu Chi Medical Foundation, Dalin, Chiayi, Taiwan; ^4^Graduate Institute of Long-Term Care, Tzu Chi University of Science and Technology, Hualien City, Hualien, Taiwan; ^5^Dalla Lana School of Public Health, University of Toronto, Toronto, Ontario, Canada

## Abstract

**Background:**

Traditional Chinese medicine (TCM) body constitution has been studied in many diseases, but few have focused on systemic lupus erythematosus (SLE) and particularly their association with disease-specific quality of life (QoL). Therefore, the aim of this study was to investigate the association of TCM body constitution and QoL in female patients with SLE.

**Methods:**

A cross-sectional study was conducted on adult female patients with a clinician-confirmed diagnosis of SLE in a regional hospital in Taiwan. TCM body constitution types were determined using the Constitution in Chinese Medicine Questionnaire (CCMQ). Disease-specific QoL of the participants was assessed using the LupusQoL. Multiple linear regression analyses were conducted to assess the associations between TCM body constitution types with the score of each of the eight domains of LupusQoL and between the numbers of multiple unbalanced body constitution types and score of each of the eight domains of LupusQoL.

**Results:**

Of the 317 female patients with SLE, 22 (6.9%) were classified to have a gentleness balanced body constitution type. Among the remaining 295 patients with unbalanced body constitution types, Qi-deficiency was the most common (64.4%), followed by Yin-deficiency (57.6%). Multiple linear regression analyses showed that Qi-deficiency was significantly associated with the emotional, pain, and fatigue domains of the LupusQoL, whereas Yin-deficiency was significantly associated with the emotional and fatigue domains of the LupusQoL. In addition, all domains of the LupusQoL showed a general pattern of poorer QoL with increasing numbers of unbalanced body constitution types.

**Conclusions:**

Different TCM body constitution types were significantly associated with various domains of the LupusQoL. A high prevalence of multiple body constitution types in patients with SLE was observed. A consistent pattern of poorer LupusQoL with increasing numbers of unbalanced body constitution types was evident.

## 1. Introduction

Traditional Chinese medicine (TCM) is a system of medicine embedded in a complex conceptual framework that guides health maintenance and disease treatment. According to the TCM theory, both innate and acquired factors can contribute to TCM body constitution (i.e., *ti-zhi*) of individuals, which in turn can determine their disease susceptibility and development [1]. To ensure a consistent and an objective measurement of TCM body constitution for research applications, standardized questionnaires have been devised to meet this purpose, such as the Body Constitution Questionnaire (BCQ) [2] and the Constitution in Chinese Medicine Questionnaire (CCMQ) [3].

Previous studies have investigated the association of TCM body constitution with various diseases and conditions, such as coronary artery disease [4], type 2 diabetes [5], metabolic syndrome [5], IgA nephropathy [6], obesity [7], and cancer-related fatigue [8]. In addition, a recent review study based on five electronic literature databases, in both English and Chinese, identified 1639 clinical studies that evaluated the association between TCM body constitutions and various diseases. A total of 19 disease categories and 333 different diseases were included, with hypertension, diabetes, stroke, coronary atherosclerotic heart disease, sleep disorders, breast cancer, dysmenorrhea, fatty liver disease, chronic viral hepatitis B, and dyslipidemia being the ten most commonly studied diseases and conditions [9].

Although TCM body constitution has been extensively studied in many diseases [10], little research has focused on patients with systemic autoimmune diseases, in particular systemic lupus erythematosus (SLE). SLE is a chronic systemic autoimmune disease that predominantly affects women of childbearing age [11]. The presentation and the course of SLE are highly variable, characterized by remissions and exacerbations. Despite advancements in the disease management of SLE, patients living with SLE have a poor health-related quality of life (QoL) compared with not only healthy people, but those with other chronic diseases, such as diabetes, hypertension, and heart failure [12, 13]. Nevertheless, few studies have examined the association of health-related QoL and body constitution, except two studies on patients with type 2 diabetes [14] and community-dwelling people [15]. Therefore, the aim of this cross-sectional study was to investigate the association between TCM body constitution and different aspects of QoL in patients with SLE.

Furthermore, according to the TCM theory, an individual can possess a body constitution of only a single type or a combination of types. However, to the best of our knowledge, previous research typically assumed the absence of multiple types of body constitution and assigned each individual to have one and only one type of body constitution. Under this assumption, different types of body constitution were often evaluated in multiple regression models as if they were independent. However, this assumption is inconsistent with patterns observed in clinical settings. Therefore, a novel approach was taken in this study, which acknowledged the presence of multiple body constitution types. Combinations of body constitution types were created, and their associations with various domains of disease-specific QoL were explored in this study.

## 2. Materials and Methods

### 2.1. Study Design and Participants

This cross-sectional study was conducted at the rheumatology outpatient clinic in a regional teaching hospital in southern Taiwan from April 2019 to August 2019. All participants signed informed consent under a study protocol approved by the institutional review board of Dalin Tzu Chi Hospital, Buddhist Tzu Chi Medical Foundation (no. B10801017). According to G∗Power software (version 3.1.9.4) [16], a sample size of 307 participants would be required to detect an effect size of 0.09 for multiple regression analysis with 20 predictors, an alpha of 0.05, and a power of 90%. The effect size was based on Cohen's *f*^2^ for multiple regression. We chose 0.09 as our effect size, which is halfway between a small (0.02) and medium (0.15) effect size as suggested by Cohen [17].

All patients at the study clinic during the study period were consecutively assessed for eligibility for enrollment in the study. Female patients aged 20 years and older, with a clinician-confirmed diagnosis of SLE based on the 1997 update of the 1982 American College of Rheumatology (ACR-97) [18] or the 2012 Systemic Lupus International Collaborating Clinics Classification Criteria (SLICC-12) [19], were included in the study. The ACR-97 is based on 11 criteria, with SLE defined as the presence of at least 4 of them serially or simultaneously, during any interval of observation. In contrast, the SLICC-12 contained 17 domains, and patients need to fulfill a minimum of four criteria, with at least one clinical criterion and one immunologic criterion.

### 2.2. Classification of Traditional Chinese Medicine Body Constitution Types

Types of TCM body constitution of the participants were determined based on the Constitution in Chinese Medicine Questionnaire (CCMQ), developed by Wang and his research team [3, 20]. The CCMQ was devised based on the constitutional theory of TCM proposed in the 1970s and was subsequently accepted in 2009 as the national standard of body constitution classification in China. In this study, we used the CCMQ (Hong Kong version), written in traditional Chinese characters, which is based on the CCMQ_60 China Standard version 1.0. Permission for the use of the CCMQ was obtained from the copyright owner Professor Wang Qi through the questionnaire developer of the Hong Kong version in traditional Chinese characters, Professor Wendy Wong at the Chinese University of Hong Kong [21].

The CCMQ consists of 60 Likert response-type items of 1 to 5 representing never, rarely, sometimes, often, and always. Based on a standard scoring algorithm proposed in the original CCMQ [1, 22], an individual can be classified into one or more of the nine types of body constitution. The nine types of body constitution include one type of balanced constitution (gentleness) and eight types of unbalanced constitutions: Qi-deficiency, Yang-deficiency, Yin-deficiency, phlegm-dampness, damp-heat, blood stasis, Qi stagnation, and special diathesis (or inherited special). Briefly, if the score for the balanced constitution ≥60 and all the remaining eight unbalanced constitution score <40, then a balanced constitution was defined. Otherwise, if the score for balanced constitution <60 and Qi-deficiency scores ≥40, then Qi-deficiency constitution was defined, and the determination for the other seven unbalanced constitutions was similarly defined. For individuals who could not be categorized into any body constitution types based on the above algorithm, their body constitutions were assigned as the one with the highest score. A description of typical clinical manifestations of the nine types of body constitution can be found in a review article by Sun et al. [1].

A validation study of the Hong Kong version of the CCMQ showed that the internal reliability Cronbach's *α* was 0.89 and the intraclass correlation for two-week test-retest reliability ranged from 0.71 to 0.88. Confirmatory factor analysis was able to reproduce the 9-factor structure as the original CCMQ. In addition, no significant flooring or ceiling effect was observed in the CCMQ [21].

### 2.3. Measurement of Disease-Specific Quality of Life

Disease-specific QoL of the study participants was assessed using the LupusQoL scale [23]. The LupusQoL consists of 34 items grouped into eight domains of QoL, including physical health (8 items), emotional health (6 items), body image (5 items), pain (3 items), planning (3 items), fatigue (4 items), intimate relationships (2 items), and burden to others (3 items). A four-week recall period was used. The response scale was in a five-point Likert format, where 0 = all of the time, 1 = most of the time, 2 = a good bit of the time, 3 = occasionally, and 4 = never. The mean raw scores were transformed to a scale of 0 to 100 points. A higher score in a domain indicates a better QoL for that particular domain [24]. We used the official Chinese for Taiwan version of the LupusQoL, and permission for use was obtained from RWS Life Sciences (http://www.corptransinc.com/sites/lupusqol/home). A study on 208 patients with SLE in China, using the LupusQoL-China culturally adapted from the Chinese for Taiwan version, demonstrated evidence of construct validity when compared with equivalent domains on the EQ-5D. The internal consistency reliability Cronbach's *α* ranged from 0.81 to 0.96 with the test-retest reliability ranging from 0.84 to 0.97 across different domains of the LupusQoL [25].

### 2.4. Measurement of Demographic and Clinical Variables

The CCMQ, LupusQoL, and other demographic and clinical information of the patients were ascertained using a paper-based questionnaire with the assistance of two experienced research nurses of the rheumatology outpatient clinic. The demographic and clinical information included sex, age interval, body mass index, educational level, marital status, job change due to SLE, employment status, self-perceived health status, duration of SLE, age of diagnosis of SLE, alcohol use, smoking, regular exercise, and sleep duration.

SLE disease activity was assessed using 24-item Systemic Lupus Erythematosus Disease Activity Index 2000 (SLEDAI-2K) [26]. The recall period for disease activity was the previous 10 days. The score ranges from 0 to 105 points, with higher values signifying greater disease activity.

### 2.5. Statistical Analysis

All statistical analyses were performed using IBM SPSS Statistics for Windows, Version 25.0.0.2 (IBM Corp., Armonk, NY, USA). All statistical tests were two-tailed with a level of significance set at 0.05. Continuous variables were summarized as mean with standard deviation (SD), and categorical variables were presented as frequencies and percentages.

Multiple linear regression analyses were performed separately for each of the eight domains of the LupusQoL as the dependent variable. Body constitution was analyzed in two different approaches. First, it was analyzed conventionally where each type of body constitution was considered independent of other types of body constitution. In other words, the effect of correlations of different types of body constitution in patients with multiple types of body constitution is assumed to be absent. In addition, the potential confounding effects of other demographic and clinical variables were evaluated in the regression model using a stepwise variable selection procedure. These variables included age interval, body mass index, educational level, marital status, change of job due to SLE, employment status, self-perceived health status, duration of SLE, alcohol use, smoking, regular exercise, sleep duration, and SLEDAI-2K.

Second, new variables representing multiple types of body constitution were created based on combinations of the eight unbalanced body constitution types. In addition to the eight types of single body constitution (_8_*C*_1_ = 8), a total of 247 possible combinations (_8_*C*_2_ = 28, _8_*C*_3_ = 56, _8_*C*_4_ = 70, _8_*C*_5_ = 56, _8_*C*_6_ = 28, _8_*C*_7_ = 8, and _8_*C*_8_ = 1) are theoretically possible with eight types of body constitution. The formula to compute the number of body constitution combinations is *n*!/*k*! (*n *−* k*)!, where *n* is the total number of body constitutions (8) and *k* is the number of selected body constitution (1 to 8, represented by the alphabet A, B, C, D, E, F, G, and H). For example, there are 28 possible different combinations of body constitution consisting of two types, which include AA, AB, AC, AD, AE, AF, AG, AH, BC, BD, BE, BF, BG, BH, CD, CE, CF, CG, CH, DE, DF, DG, DH, EF, EG, EH, FH, and GH.

All observed combinations of body constitution in the patients were first identified and grouped into one to eight types of unbalanced body constitution types. Multiple linear regression analyses were performed with each of the eight LupusQoL domains as the dependent variable. The main independent variable was a categorical variable with nine levels representing the number of unbalanced body constitutions (1 to 8). Patients with no unbalanced body constitution were set as the reference category. The same set of potential confounding variables listed in the aforementioned multiple regression analysis was evaluated using the stepwise variable selection procedure.

For all regression analyses, the linearity assumption was visually inspected using a plot of standardized residuals against standardized predictor values. The independence of errors assumption was tested using Durbin-Watson statistics. The presence of multicollinearity was evaluated using the variance inflation factor (VIF). Moreover, the effect sizes of the regression coefficients for the body constitution were quantified using partial eta squared (*η*_*p*_^2^). Values of 0.01, 0.06, and 0.14 were interpreted as small, medium, and large effects, respectively [17].

To further explore the relationship of the unbalanced body constitution types, VOSviewer version 1.6.13 for Microsoft Windows (Centre for Science and Technology Studies, Leiden University, The Netherlands) [27] was used to construct a bibliometric map for the visualization of co-occurrence of unbalanced body constitution types. We used the PubMed database format and entered the unbalanced body constitution of each patient as if they were keywords of an article (field name: OT). In other words, a citation database of 295 “articles” representing 295 patients was constructed. Co-occurrence with full counting method was used. The minimum number of occurrences of a keyword was set to two.

## 3. Results

Table 1 shows the demographic and clinical characteristics of the 317 female patients with SLE. Of them, 124 (39.1%) were between the ages of 20 to 39 years, and 167 (52.7%) had a normal body mass index. Regarding the self-perceived health status, 51 (16.1%) reported poor or very poor. The score of LupusQoL ranged from 71.5 for the fatigue domain to 83.3 for the emotional health domain.

The distribution of TCM body constitution types in patients with SLE is shown in Table 2. Of the 317 patients, 22 (6.9%) were classified to have the gentleness balanced body constitution. Among the 295 patients with unbalanced body constitution types, Qi-deficiency was the commonest type (64.4%), followed by Yin-deficiency (57.6%), blood-stasis (48.5%), Yang-deficiency (39.3%), phlegm-wetness (36.9%), Qi-depression (32.2%), wetness-heat (28.5%), and special diathesis (22.7%).

Because body constitution with more than one type is commonly observed in clinical settings, we further classified our patients according to the number of unbalanced body constitution types (Table 3). Despite having just one unbalanced body constitution was the most common condition (26.1%), 218 patients (73.9%) were found to have two to eight types of unbalanced body constitution. Two to three body constitution types accounted for over a third of all the patients. Table 3 also shows the proportion of observed combinations over all possible combinations for patients with one to eight types of unbalanced body constitution. For patients with two unbalanced body constitutions, 75.0% of all possible combinations were observed in our patients, with Qi-deficiency and Yin-deficiency being the most common combination. For patients with three unbalanced body constitutions, 39.3% of all possible combinations were observed in our patients, with Qi-deficiency, Yin-deficiency, and phlegm-wetness being the most common combination.

**Figure 1:**
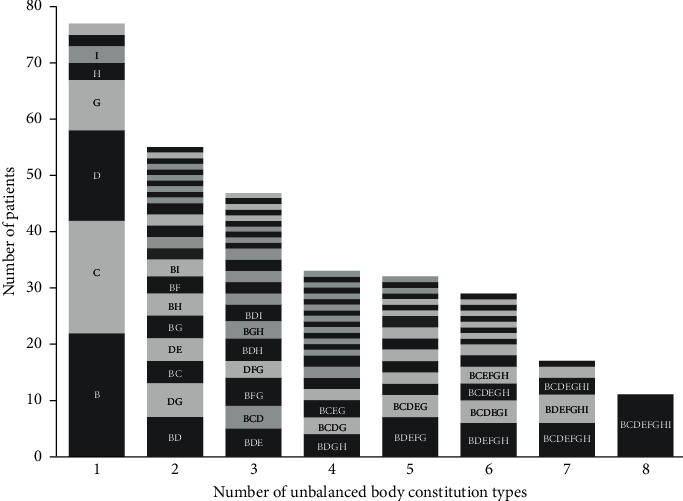
Distribution of body constitution in patients with a single unbalanced body constitution and those with multiple unbalanced body constitution types. B: Qi-deficiency; C: Yang-deficiency; D: Yin-deficiency; E: phlegm-wetness; F: wetness-heat; G: blood-stasis; H: Qi-depression; and I: special diathesis. For clarity, only those body constitution types with at least 3 patients were shown in the figure.

**Figure 2:**
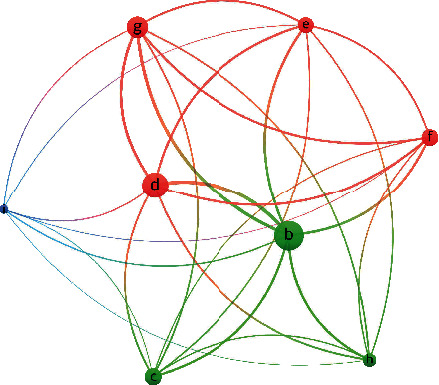
Visualization on co-occurrence unbalanced body constitution types. Three clusters were observed including (1) Qi-deficiency (b) with Yang-deficiency (c) and Qi-depression (h); (2) Yin-deficiency (d) with blood-stasis (g), wetness-heat (f), and phlegm-wetness (e); and (3) special diathesis (i).

Figure 1 is a stacked bar graph showing the pattern of various combinations of unbalanced body constitution types for different numbers of unbalanced body constitution types. For clarity, only those combinations with at least three individuals are shown in the figure. In addition, Figure 2 depicts the co-occurrence of unbalanced body constitution types in our patients with SLE. Three clusters emerged, which include cluster 1 linking Qi-deficiency, Yang-deficiency, and Qi-depression; cluster 2 linking Yin-deficiency, blood-stasis, wetness-heat, and phlegm-wetness; and cluster 3 with just special diathesis. In a co-occurrence diagram, the size of the circle of an item represents its relative weight. It can be seen that Qi-deficiency was the most important body constitution in cluster 1, whereas Yin-deficiency was the most important one in cluster 2. In addition, the distance between two circles reflects the strength of the relationship between them, with a shorter span representing a stronger connection. Although Qi-deficiency and Yin-deficiency belonged to two different clusters, they were connected by a thick line, which indicated that there was also a high density of co-occurrence between them. This observation is consistent with the pattern shown in Figure 1 where Qi-deficiency and Yin-deficiency often exist together in patients with combination types of body constitution.

The associations between each of the eight domains of LupusQoL and body constitution types were analyzed in two different ways. First, the typical multivariable regression approach was used where all body constitution types were simultaneously evaluated during model development (Table 4). Yang-deficiency (*P*=0.021) and phlegm-wetness (*P*=0.015) were significantly associated with a lower score (worse condition) in the physical health domain. Qi-deficiency (*P*=0.014), Yang-deficiency (*P*=0.042), Yin-deficiency (*P*=0.008), wetness-heat (*P*=0.006), and Qi-depression (*P* < 0.001) were significantly associated with a lower score in the emotional health domain. Phlegm-wetness (*P* < 0.001), wetness-heat (*P*=0.036), and Qi-depression (*P* < 0.001) were significantly associated with a low score in the body image domain. Qi-deficiency (*P*=0.004), phlegm-wetness (*P*=0.007), and blood-stasis (*P* < 0.001) were significantly associated with a lower score in the pain domain. Yang-deficiency (*P*=0.005) was significantly associated with a lower score in the planning domain. Qi-deficiency (*P* < 0.001), Yang-deficiency (*P*=0.012), Yin-deficiency (*P*=0.022), phlegm-wetness (*P* < 0.001), blood-stasis (*P*=0.037), and Qi-depression (*P*=0.008) were significantly associated with a lower score in the fatigue domain. Yang-deficiency (*P*=0.004), blood-stasis (*P*=0.015), and Qi-depression (*P*=0.010) were significantly associated with a lower score in the intimate relationships domain. Wetness-heat (*P* < 0.001) and special diathesis (*P*=0.029) were significantly associated with a lower score in the burden to others domain. In addition, gentleness body constitution type was not significantly associated with any of the eight domains of LupusQoL.

According to the criteria proposed by Cohen [17], only two of the 24 significant associations had an *η*_*p*_^2^ above the cut-off value of 0.06 for a medium effect size. *η*_*p*_^2^ for the association between Qi-depression and emotional health QoL domain was 0.102, and that between wetness-heat and burden to others QoL domain was 0.073. The magnitude of *η*_*p*_^2^ for the remaining 22 associations was between 0.013 and 0.042.

**Figure 3:**
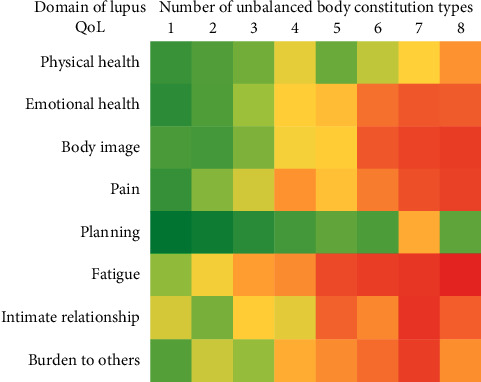
A heat map of the number of unbalanced body constitution type and the domain of LupusQoL. The color scale ranges from green (highest score in LupusQoL) to yellow, orange, and then red (lowest score in LupusQoL).

The second approach in the data analysis used the number of multiple unbalanced body constitution types in each patient as a predictor to evaluate their association with each of the eight domains of LupusQoL (Table 5). A general pattern of increasing absolute magnitude in the regression coefficients with a higher number of unbalanced body constitution type was observed. Compared with patients who had no unbalanced body constitution, those with an unbalanced body constitution of one to eight types showed significantly lower scores in the fatigue QoL domain, ranging from −9.59 in those with unbalanced body constitution of only one type to −47.71 in those with an unbalanced body constitution consisting of eight types. In addition, only patients with an unbalanced body constitution consisting of seven types were significantly associated with the planning QoL domain (*P*=0.025). For the remaining six QoL domains, patients with an unbalanced body constitution consisting of three or more types were significantly associated with the emotional health and pain QoL domains. Patients with an unbalanced body constitution consisting of four or more types were significantly associated with the body image and burden to others QoL domains. Moreover, patients with an unbalanced body constitution consisting of five or more types were significantly associated with the intimate relationships QoL domain. In addition, patients with an unbalanced body constitution consisting of four types and six to eight types were significantly associated with the physical health QoL domain.  Figure 3 is a heat map to visually express the regression coefficients shown in Table 5. A clear overall pattern of decreased QoL was evident.

Regarding the effect size shown in Table 5, *η*_*p*_^2^ for the association between an unbalanced multiple body constitution consisting of six types and fatigue QoL domain was the largest (0.136) one of the 39 significant associations, with a magnitude close to the criteria of a large effect size. In addition, eight other associations showed a medium effect size, ranging from an *η*_*p*_^2^ of 0.060 for the association between a multiple unbalanced body constitution consisting of eight types and the pain QoL domain to an *η*_*p*_^2^ of 0.129 for the association between a multiple unbalanced body constitution consisting of eight types and the fatigue QoL domain.

## 4. Discussion

According to the TCM theory, the human body consists of a number of mutually intertwining physiological systems. A lack of harmony of the interaction between these systems can manifest as physical and emotional symptoms and can lead to increased susceptibility to disease when the imbalance is severe. TCM body constitution classification is a typical approach to determine the condition and balance of these systems. In the present study, the CCMQ was used to classify female patients with SLE into nine body constitution types and to evaluate the association between disease-specific QoL with eight unbalanced body constitution types. Two unique aspects of this study should be mentioned. First, this is the first investigation of the association of disease-specific QoL and body constitution types in female patients with SLE. The results showed that Qi-deficiency was the most common unbalanced body constitution (64.4%) in our patients. This finding is in line with the results of a cross-sectional study of 1220 participants with varied health status. It was found that the prevalence of Qi-deficiency syndrome in individuals classified as healthy, subhealthy, and with chronic diseases was 11.5%, 26.2%, and 57.6%, respectively [28]. A large-scale epidemiological study conducted on 21948 individuals in China showed that 13.4% of them had a Qi-deficiency body constitution [29]. Moreover, our results showed that Qi-deficiency was significantly associated with the emotional, pain, and fatigue domains of the LupusQoL. Persistent fatigue, lack of strength, dizziness, and not feeling like talking are common symptoms associated with Qi-deficiency. A study on 198 patients with cancer also showed that those with Qi-deficiency had significantly poorer QoL in physical, psychological, and social domains [30].

The second most prevalent unbalanced body constitution type observed in this study was Yin-deficiency (57.6%), which is consistent with the TCM theory that the primary pathogenic change of SLE is Yin-deficiency [31]. Our results also showed that Yin-deficiency was significantly associated with the emotional and fatigue domains of the LupusQoL, which is in agreement with the common clinical manifestations of anxiety, irritability, proneness to emotional disturbance, and constant fatigue in patients with Yin-deficiency [31]. A study on 705 patients with type 2 diabetes reported that those with Yin-deficiency had significantly poorer health-related QoL in all eight domains of the Short Form 36 [14].

Findings from this study also showed that Qi-deficiency and Yin-deficiency appeared to be the most common type of body constitution combination. This combination is often clinically observed in patients with SLE, and it has been used as one of the inclusion criteria for patient selection in a randomized controlled pilot study of the Chinese herbal formula Zi Shen Qing for the treatment of SLE [32]. It is considered in TCM that the root cause of SLE is Yin-deficiency, and as the disease progresses, Qi-deficiency, Yang-deficiency, blood-stasis, and wetness-heat may successively appear and exist together [33].

Based on a network diagram of co-occurrence, three clusters of unbalanced body constitution emerged. The first cluster linked Qi-deficiency to Yang-deficiency and Qi-depression, whereas the second cluster linked Yin-deficiency, blood-stasis, wetness-heat, and phlegm-wetness. It could be inferred that the first cluster represented individuals with lassitude, tiredness, cold hands and feet, anxiety, depression, and pale complexion, whereas the second cluster represented those with feverish sensation in the palms and soles and greasy complexion. Nevertheless, given all the connecting lines among the different body constitution types, it is clear that the existence of various combinations of unbalanced body constitution types in a single person is a rule rather than an exception. This observation is compatible with the theory of TCM and clinical experience. In a cross-sectional study of 1084 patients attending outpatient clinics in Hong Kong, 65% of the patients had more than one unbalanced body constitution types [21]. In our study, a higher proportion of 73.9% among our patients with SLE was found to have more than one unbalanced body constitution type.

As multiple unbalanced body constitution types are common, it is of interest to explore whether individuals with more types were associated with a poorer QoL. In fact, a unique aspect of this study is the acknowledgement of the presence of multiple TCM body constitution types in an individual. In the past, different body constitution types are typically treated as variables that are independent of each other. In other words, all body constitution types are entered into a multivariable regression model as predictors to determine their independent effects on the outcome variable. For example, using multiple logistic regression, a study of 306 patients with impaired glucose regulation showed that phlegm-damp or damp-heat constitution, measured by the Body Constitution Questionnaire (BCQ) [2], was significantly and independently associated with a higher risk of diabetes [34]. In another study of 3748 community-dwelling individuals, multinomial logistic regression analysis was performed to assess the associations between TCM body constitution and outcomes of overweight, obesity, and underweight [7]. Again, these statistical models assumed that the effects of each of the body constituent types on the outcome variables are statistically independent from each other. In contrast, the present study used the number of types of body constitution as predictors of QoL. Our results indicated that except for the planning domain, all other domains of the LupusQoL showed a consistent pattern of poorer QoL with increasing numbers of unbalanced body constitution types. The increasing pattern was particularly prominent in the fatigue domain. Moreover, the effect sizes in the fatigue domain were the largest among all the regression coefficients. This finding has important clinical implications for TCM management of SLE in that the treatment principle in patients with various combinations of multiple body constitution types could be different. For example, to prevent the progression of SLE in patients with Qi-deficiency, Yin-deficiency, and phlegm-wetness body constitution, simply nourishing Yin is not sufficient. The excess symptoms caused by phlegm-wetness should first be resolved before treating the primary condition [7]. Regarding research implications, future studies on TCM body constitution should explore common combinations of body constitution types in addition to treating each body constitution type separately as in the past.

A few limitations of this study should be mentioned. First, this study used a cross-sectional design, which precluded the assessment of changes in body constitution and QoL with disease progression. Second, the study participants were recruited from a single regional teaching hospital in southern Taiwan and thus might limit the generalizability of the results to other settings. Third, patients with severe complications were not included in this study, which might also affect the generalizability of the results. Fourth, the exact combinations of the body constitution type that most severely affected QoL could not be identified with the current sample size due to the small number of patients in each combination of body constitution types.

## 5. Conclusion

SLE is a complex and clinically heterogeneous disease. Findings from this cross-sectional study showed that different TCM body constitution types were significantly associated with physical health, emotional health, body image, pain, planning, fatigue, intimate relationships, and burden to others domains of the LupusQoL. In addition, a high prevalence of multiple unbalance body constitution types in patients with SLE was observed, and a consistent pattern of poorer LupusQoL with increasing numbers of unbalanced body constitution types, particularly in the fatigue domain, was evident. Future studies and clinical practice should take into account the presence of various combinations of body constitution types.

## Figures and Tables

**Table 1:** Demographic and clinical characteristics of patients with systemic lupus erythematosus (*N* = 317).

Variable	*n*	(%)
Age interval (years)		
20–29	39	(12.3)
30–39	85	(26.8)
40–49	77	(24.3)
50–59	65	(20.5)
≥60	51	(16.1)

Body mass index (kg/m^2^)		
Normal (≥18.5 and <24.0)	167	(52.7)
Underweight (<18.5)	45	(14.2)
Overweight (≥24 and <27)	60	(18.9)
Obese (≥27)	45	(14.2)

Educational level		
Junior high school or below	57	(18.0)
Senior high school or college	142	(44.8)
University or above	118	(37.2)

Marital status		
Single	106	(33.4)
Being married, widowed, divorced	211	(66.6)

Change job due to SLE		
No	223	(70.3)
Yes	94	(29.7)

Employment status		
Unemployed	117	(36.9)
Employed	200	(63.1)

Self-perceived health status		
Good or very good	83	(26.2)
Average	183	(57.7)
Poor or very poor	51	(16.1)

Disease duration (years)		
≤9	108	(34.1)
10–15	98	(30.9)
≥16	111	(35.0)

Alcohol use		
No	248	(78.2)
Yes	69	(21.8)

Smoking		
No	296	(93.4)
Yes	21	(6.6)

Regular exercise (at least several times a week)		
No	169	(53.3)
Yes	148	(46.7)

Sleep duration (hours)		
≤5	63	(19.9)
6–7	195	(61.5)
≥8	59	(18.6)
SLEDAI-2K, mean (SD)	5.44	(7.10)

Domain of LupusQoL		
Physical health	81.1	(20.1)
Emotional health	83.3	(19.4)
Body image	82.5	(26.7)
Pain	80.0	(26.7)
Planning	80.8	(23.6)
Fatigue	71.5	(29.8)
Intimate relationships	74.0	(33.0)
Burden to others	72.3	(23.6)

SD: standard deviation; SLEDAI-2K: Systemic Lupus Erythematosus Disease Activity Index 2000.

**Table 2:** Distribution of traditional Chinese medicine body constitutions in female patients with systemic lupus erythematosus (*N* = 317).

Body constitution type	*n* (% of *N*)	% of unbalanced body constitution type (*n* = 295)
Balanced		
Gentleness	22 (6.9)	Not applicable

Unbalanced		
Qi-deficiency	190 (59.9)	64.4
Yang-deficiency	116 (36.6)	39.3
Yin-deficiency	170 (53.6)	57.6
Phlegm-wetness	109 (34.4)	36.9
Wetness-heat	84 (26.5)	28.5
Blood-stasis	143 (45.1)	48.5
Qi-depression	95 (30.0)	32.2
Special diathesis	67 (21.1)	22.7

The total percentage exceeded 100 because of the presence of multiple body constitutions in some patients.

**Table 3:** Number of unbalanced traditional Chinese medicine body constitutions in female patients with systemic lupus erythematosus (*N* = 295).

Number of unbalanced body constitution types	*n* (%)	Cumulative *n* (%)	Observed combination/all possible combinations (%)	Type with the highest frequency (count)
1	77 (26.1)	77 (26.1)	8/8 (100.0)	B (22)
2	53 (18.0)	130 (44.1)	21/28 (75.0)	BD (7)
3	47 (15.9)	177 (60.0)	22/56 (39.3)	BDE (5)
4	33 (11.2)	210 (71.2)	22/70 (31.4)	BDGH (4)
5	31 (10.5)	241 (81.7)	15/56 (26.8)	BDEFG (7)
6	26 (8.8)	267 (90.5)	12/28 (42.9)	BDEFGH (6)
7	17 (5.8)	284 (96.3)	5/8 (62.5)	BCDEFGH (6)
8	11 (3.7)	295 (100.0)	1/1 (100.0)	BCDEFGHI (11)
Total	—	—	108/255 (42.4)	—

The total number of patients in this study was 317, which is equal to the 295 patients shown in Table 2 plus 22 patients with a balanced body constitution (gentleness type). B: Qi-deficiency; C: Yang-deficiency; D: Yin-deficiency; E: phlegm-wetness; F: wetness-heat; G: blood-stasis; H: Qi-depression; and I: special diathesis.

**Table 4:** Multiple linear regression analyses of body constitution type for each of the eight domains of the LupusQoL.

Domains of LupusQoL	Type of body constitution								
	Adjusted beta coefficient (95% confidence interval) [*P* value] and partial eta squared								
	Gentleness	Qi-deficiency	Yang-deficiency	Yin-deficiency	Phlegm-wetness	Wetness-heat	Blood-stasis	Qi-depression	Special diathesis
Physical health	—	—	−4.64 (−8.56,−0.71) [0.021] *η*_*p*_^2^ = 0.017	—	−4.91 (−8.84,−0.97) [0.015] *η*_*p*_^2^ = 0.019		—	—	—

Emotional health	—	−4.84 (−8.71, −0.98) [0.014] *η*_*p*_^2^ = 0.020	−3.83 (−7.52, −0.14) [0.042] *η*_*p*_^2^ = 0.013	−4.93 (−8.57, −1.29) [0.008] *η*_*p*_^2^ = 0.023	—	−6.00 (−10.26, −1.75) [0.006] *η*_*p*_^2^ = 0.025	—	−12.94 (−17.26,−8.62) [<0.001] *η*_*p*_^2^ = 0.102	—

Body image	—	—	—	—	−11.10 (−16.63, −5.57) [<0.001] *η*_*p*_^2^ = 0.050	−6.26 (−12.10, −0.43) [0.036] *η*_*p*_^2^ = 0.015	—	−10.37 (−16.11,−4.63) [<0.001] *η*_*p*_^2^ = 0.041	—

Pain	—	−8.14 (−13.69, −2.59) [0.004] *η*_*p*_^2^ = 0.026	—	—	−7.82 (−13.44, −2.20) [0.007] *η*_*p*_^2^ = 0.024	—	−9.52 (−15.00, −4.03) [<0.001] *η*_*p*_^2^ = 0.036	—	—

Planning	—	—	−8.00 (−13.56, −2.45) [0.005] *η*_*p*_^2^ = 0.025	—	—	—	—	—	—

Fatigue	—	−8.44 (−13.26,−3.61) [<0.001] *η*_*p*_^2^ = 0.037	−5.82 (−10.38,−1.26) [0.012] *η*_*p*_^2^ = 0.020	−5.30 (−9.83,−0.76) [0.022] *η*_*p*_^2^ = 0.017	−9.52 (−14.64, −4.40) [<0.001] *η*_*p*_^2^ = 0.042	—	−4.97 (−9.64,−0.30) [0.037] *η*_*p*_^2^ = 0.014	−7.18 (−12.48,−1.87) [0.008] *η*_*p*_^2^ = 0.023	—

Intimate relationships	–	—	−10.82 (−18.23, −3.42) [0.004] *η*_*p*_^2^ = 0.034	—	—	—	−9.13 (−16.49,−1.78) [0.015] *η*_*p*_^2^ = 0.025	−10.10 (−17.81,−2.39) [0.010] *η*_*p*_^2^ = 0.028	—

Burden to others	—	—	—	—	—	−17.08 (−23.90, −10.25) [<0.001] *η*_*p*_^2^ = 0.073	—	—	−8.16 (−15.50, −0.83) [0.029] *η*_*p*_^2^ = 0.015

All multiple linear regression models were adjusted, using stepwise variable selection procedure, for the variables listed in Table 1, except the domains of LupusQoL.

**Table 5:** Multiple linear regression analyses of the eight domains of the LupusQoL for 0 to 8 unbalanced body constitution types.

Domains of LupusQoL	Number of unbalanced body constitution types								
	Adjusted beta coefficient (95% confidence interval) [*P* value] and partial eta squared								
	0	1	2	3	4	5	6	7	8
Physical health	Ref	−4.44 (−12.33, 3.46) [0.270] *η*_*p*_^2^ = 0.004	−6.25 (−14.52, 2.03) [0.138] *η*_*p*_^2^ = 0.007	−8.07 (−16.56, 0.42) [0.062] *η*_*p*_^2^ = 0.011	−12.84 (−21.79, −3.88) [0.005] *η*_*p*_^2^ = 0.026	−7.69 (−16.79, 1.42) [0.098] *η*_*p*_^2^ = 0.009	−11.49 (−20.93, −2.05) [0.017] *η*_*p*_^2^ = 0.019	−14.07 (−24.63, −3.50) [0.009] *η*_*p*_^2^ = 0.022	−20.18 (−32.48, −7.88) [0.001] *η*_*p*_^2^ = 0.033

Emotional health	Ref	−3.05 (−10.78, 4.67) [0.438] *η*_*p*_^2^ = 0.002	−6.05 (−14.15, 2.06) [0.143] *η*_*p*_^2^ = 0.007	−10.25 (−18.54, −1.97) [0.015] *η*_*p*_^2^ = 0.019	−14.37 (−23.13, −5.60) [0.001] *η*_*p*_^2^ = 0.033	−15.84 (−24.75, −6.94) [<0.001] *η*_*p*_^2^ = 0.039	−25.66 (−34.90, −16.42) [<0.001] *η*_*p*_^2^ = 0.090	−30.73 (−41.08, −20.38) [<0.001] *η*_*p*_^2^ = 0.101	−30.17 (−42.29, −18.06) [<0.001] *η*_*p*_^2^ = 0.073

Body image	Ref	−5.78 (−16.02, 4.46) [0.267] *η*_*p*_^2^ = 0.004	−5.32 (−15.96, 5.33) [0.326] *η*_*p*_^2^ = 0.003	−8.78 (−19.67, 2.11) [0.114] *η*_*p*_^2^ = 0.008	−13.50 (−25.30, −1.71) [0.025] *η*_*p*_^2^ = 0.017	−14.57 (−26.25, −2.88) [0.015] *η*_*p*_^2^ = 0.020	−31.13 (−43.20, −19.06) [<0.001] *η*_*p*_^2^ = 0.080	−36.29 (−49.74, −22.83) [<0.001] *η*_*p*_^2^ = 0.087	−38.64 (−54.01, −23.28) [0.012] *η*_*p*_^2^ = 0.077

Pain	Ref	−4.06 (−14.82, 6.69) [0.458] *η*_*p*_^2^ = 0.002	−9.06 (−20.36, 2.24) [0.116] *η*_*p*_^2^ = 0.008	−12.19 (−23.76, −0.62) [0.039] *η*_*p*_^2^ = 0.014	−20.51 (−32.83, −8.19) [0.001] *η*_*p*_^2^ = 0.034	−15.52 (−27.89, −3.16) [0.014] *η*_*p*_^2^ = 0.020	−23.63 (−36.57, −10.69) [<0.001] *η*_*p*_^2^ = 0.041	−32.55 (−47.05, −18.04) [<0.001] *η*_*p*_^2^ = 0.061	−37.11 (−53.78, −20.45) [<0.001] *η*_*p*_^2^ = 0.060

Planning	Ref	2.34 (−9.13, 13.80) [0.689] *η*_*p*_^2^ = 0.001	0.41 (−11.67, 12.48) [0.947] *η*_*p*_^2^ = <0.001	−2.68 (−15.08, 9.73) [0.671] *η*_*p*_^2^ = 0.001	−5.27 (−18.38, 7.84) [0.430] *η*_*p*_^2^ = 0.002	−7.15 (−20.40, 6.10) [0.289] *η*_*p*_^2^ = 0.004	−5.93 (−19.79, 7.92) [0.400] *η*_*p*_^2^ = 0.002	−17.57 (−32.95, −2.20) [0.025] *η*_*p*_^2^ = 0.016	−7.08 (−25.05, 10.89) [0.439] *η*_*p*_^2^ = 0.002

Fatigue	Ref	−9.59 (−18.60, −0.59) [0.037] *η*_*p*_^2^ = 0.014	−13.42 (−22.87, −3.98) [0.005] *η*_*p*_^2^ = 0.025	−19.19 (−28.79, −9.60) [<0.001] *η*_*p*_^2^ = 0.049	−21.19 (−31.43, −10.96) [<0.001] *η*_*p*_^2^ = 0.052	−34.57 (−44.97, −24.16) [<0.001] *η*_*p*_^2^ = 0.124	−37.87 (−48.64, −27.09) [<0.001] *η*_*p*_^2^ = 0.136	−40.26 (−52.35, −28.18) [<0.001] *η*_*p*_^2^ = 0.124	−47.71 (−61.75, −33.67) [<0.001] *η*_*p*_^2^ = 0.129

Intimate relationships	Ref	−12.29 (−27.70, 3.11) [0.117] *η*_*p*_^2^ = 0.011	−8.46 (−24.36, 7.44) [0.295] *η*_*p*_^2^ = 0.005	−14.59 (−31.20, 2.01) [0.085] *η*_*p*_^2^ = 0.013	−12.74 (−29.89, 4.40) [0.144] *η*_*p*_^2^ = 0.009	−28.71 (−47.02, −10.40) [0.002] *η*_*p*_^2^ = 0.041	−22.18 (−40.70, −3.65) [0.019] *η*_*p*_^2^ = 0.024	−41.81 (−61.02, −22.60) [<0.001] *η*_*p*_^2^ = 0.075	−29.50 (−52.30, −6.70) [0.011] *η*_*p*_^2^ = 0.028

Burden to others	Ref	−6.52 (−19.60, 6.56) [0.327] *η*_*p*_^2^ = 0.003	−11.96 (−25.70, 1.79) [0.088] *η*_*p*_^2^ = 0.010	−9.77 (−23.74, 4.21) [0.170] *η*_*p*_^2^ = 0.006	−17.38 (−32.24, −2.52) [0.022] *η*_*p*_^2^ = 0.017	−21.23 (−36.36, −6.11) [0.006] *η*_*p*_^2^ = 0.024	−26.68 (−42.35, −11.00) [<0.001] *η*_*p*_^2^ = 0.035	−37.83 (−55.39, −20.27) [<0.001] *η*_*p*_^2^ = 0.056	−21.11 (−41.53, −0.69) [0.043] *η*_*p*_^2^ = 0.013

All multiple linear regression models were adjusted, using the stepwise variable selection procedure, for the variables listed in Table 1, except the domains of LupusQoL. Ref: reference group, i.e., patients without any unbalanced body constitution types.

## Data Availability

The data used to support the findings of this study are available from the corresponding author upon reasonable request.

## Abbreviations

ACR: American College of Rheumatology
CCMQ: Constitution in Chinese Medicine Questionnaire
HRQoL: Health-related quality of life
SD: Standard deviation
SLE: Systemic lupus erythematosus
SLEDAI-2K: Systemic Lupus Erythematosus Disease Activity Index 2000
TCM: Traditional Chinese medicine
VIF: Variance inflation factor.
